# Separate origins of the left internal and external carotid arteries from the aorta in a patient with intracerebral hemorrhage

**DOI:** 10.1016/j.radcr.2022.02.056

**Published:** 2022-03-26

**Authors:** Yuma Hiratsuka, Hideki Endo, Naoyasu Okamura, Masaaki Mikamoto, Bunsho Asayama, Kenji Kamiyama, Toshiaki Osato, Hirohiko Nakamura

**Affiliations:** Department of Neurosurgery, Nakamura Memorial Hospital, South 1, West 14, Chuo-ku, Sapporo, Hokkaido 060-8570, Japan

**Keywords:** Anomaly, Common carotid artery, Internal carotid artery, External carotid artery, Intracerebral hemorrhage

## Abstract

Agenesis of the left common carotid artery with separate origins of the left internal and external carotid arteries from the aorta is an extremely rare anomaly. This anomaly is typically asymptomatic unless associated with other conditions. We report a case of separate origins of the left internal and external carotid arteries from the aorta in a patient with intracerebral hemorrhage. A 42-year-old man was transferred to our hospital by ambulance because of left hemiparesis. Computed tomography scan revealed right putaminal hemorrhage. Computed tomography angiography and digital subtraction angiography demonstrated independent origins of the left internal carotid artery and external carotid artery from the aortic arch. Right internal carotid angiography revealed blood supply to the left anterior cerebral artery and middle cerebral artery via the anterior communicating artery. The separate origins of the left internal and external carotid arteries from the aorta may cause hemodynamic stress to the contralateral side, leading to right intracerebral hemorrhage.

## Introduction

A separate origin for the left internal and external carotid arteries from the aorta is an extremely rare anomaly. This anomaly is typically asymptomatic. We report a case of a patient with separate origins of the left internal and external carotid arteries from the aorta with right intracerebral hemorrhage.

## Case report

**Fig. 1:**
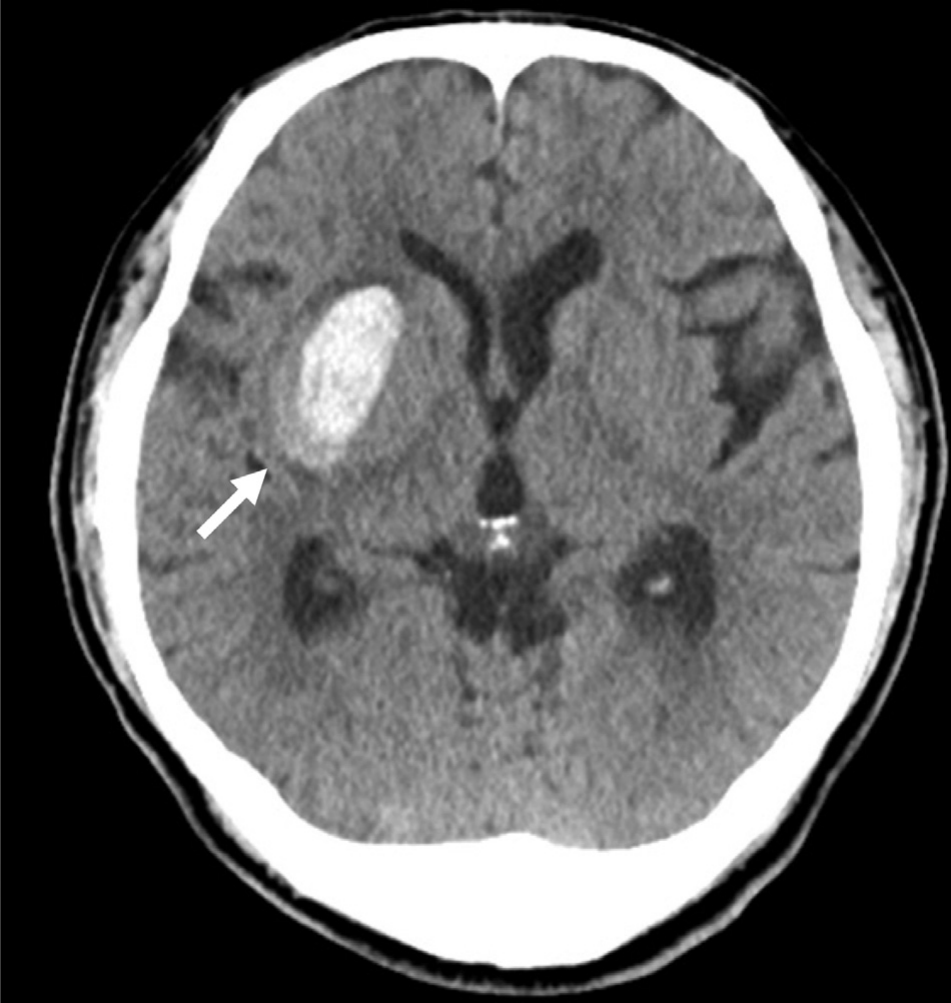
Computed tomography showing the right intracerebral hemorrhage (arrow).

**Fig. 2:**
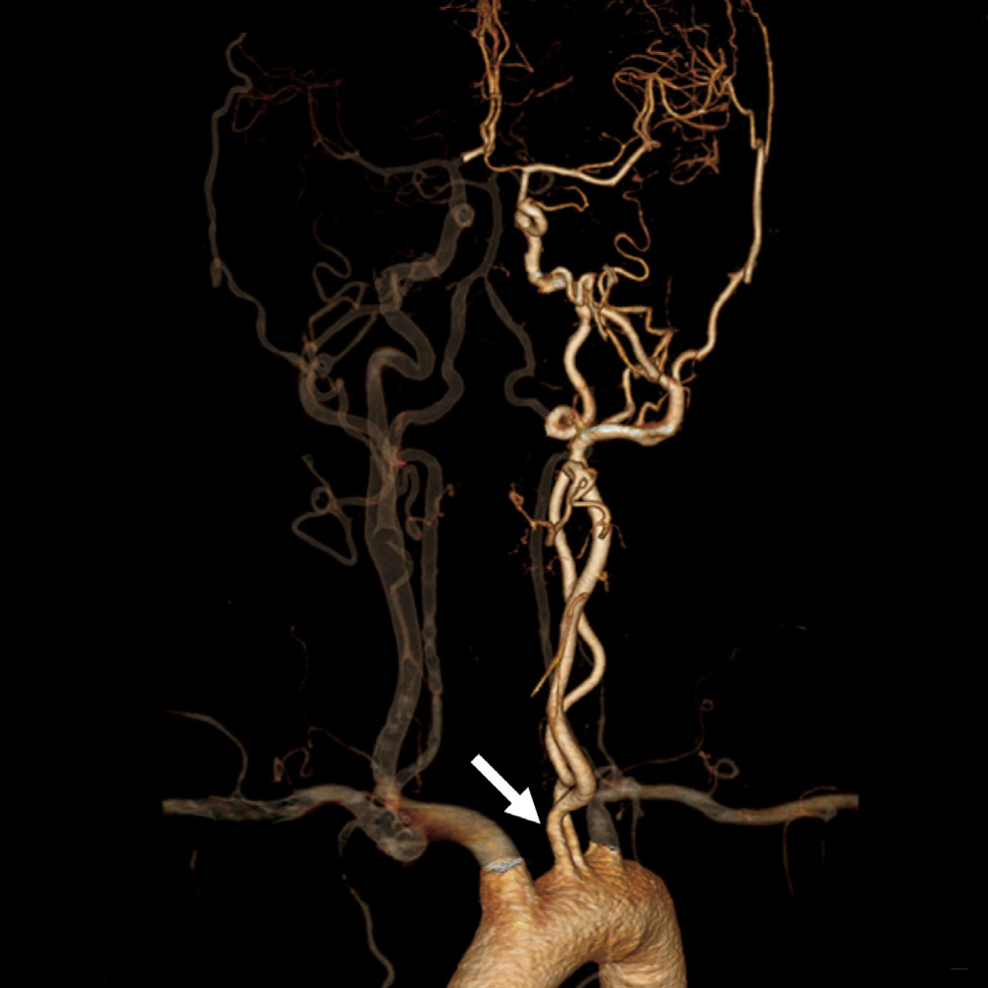
Computed tomography angiography demonstrating independent origins of the left internal and external carotid arteries from the aortic arch (arrow).

**Fig. 3:**
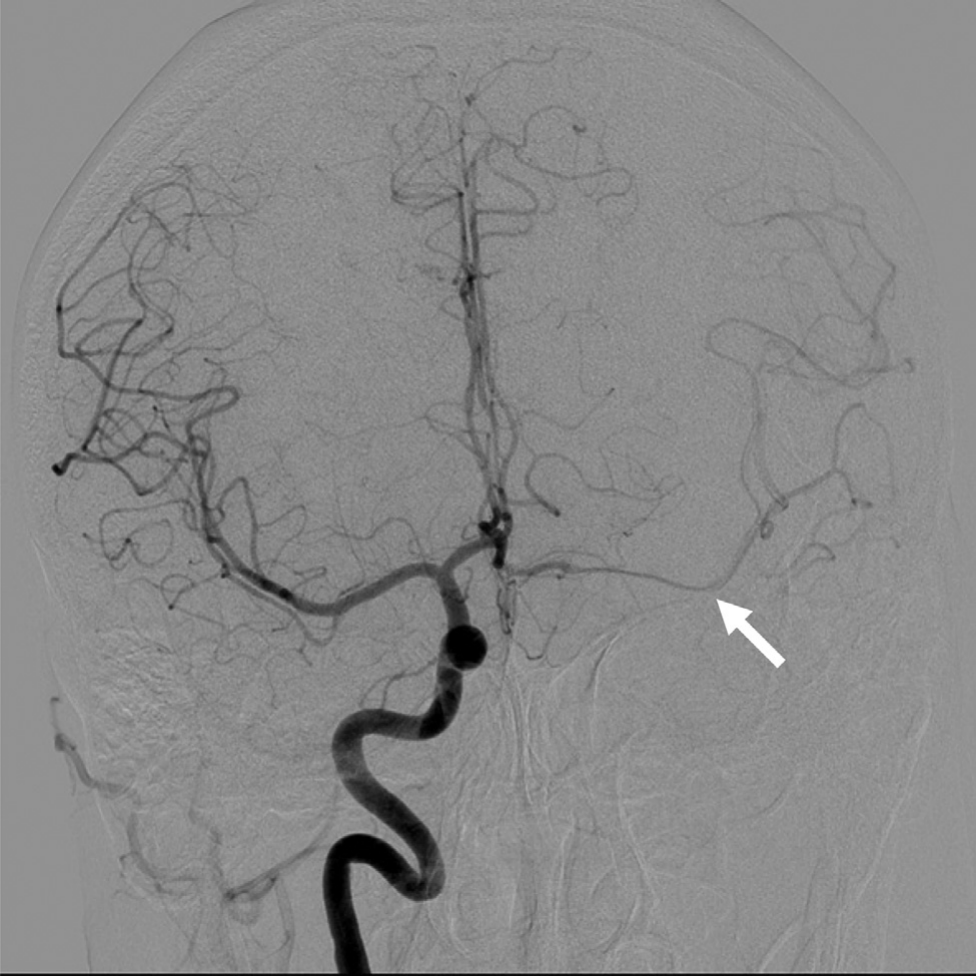
Selective right internal carotid angiogram showing blood flow on the left side via the anterior communicating artery (arrow).

A 42-year-old man was transferred to our hospital by ambulance because of left hemiparesis. The patient had been diagnosed with hypertension 2 years ago, but had not received any medical treatment. The laboratory data at admission showed diabetes mellitus. Computed tomography (CT) scan revealed right putaminal hemorrhage (Fig. 1). Bone CT showed the left carotid canal was narrower than that on the right side. CT angiography and digital subtraction angiography demonstrated independent origins of the left internal carotid artery (ICA) and external carotid artery (ECA) from the aortic arch, and the left ICA was noted to be tortuous, dysplastic, and have smaller caliber (Fig. 2). The left ECA originated proximal to the left ICA (Fig. 2). Right internal carotid angiogram revealed no obvious abnormal vessels within or around the hematoma (Fig. 3). The left anterior cerebral artery and middle cerebral artery received blood flow from the right ICA via the anterior communicating artery (Fig. 3). We performed conservative therapy, particularly antihypertensive treatment. He was discharged without any permanent deficit.

## Discussion

**Fig. 4:**
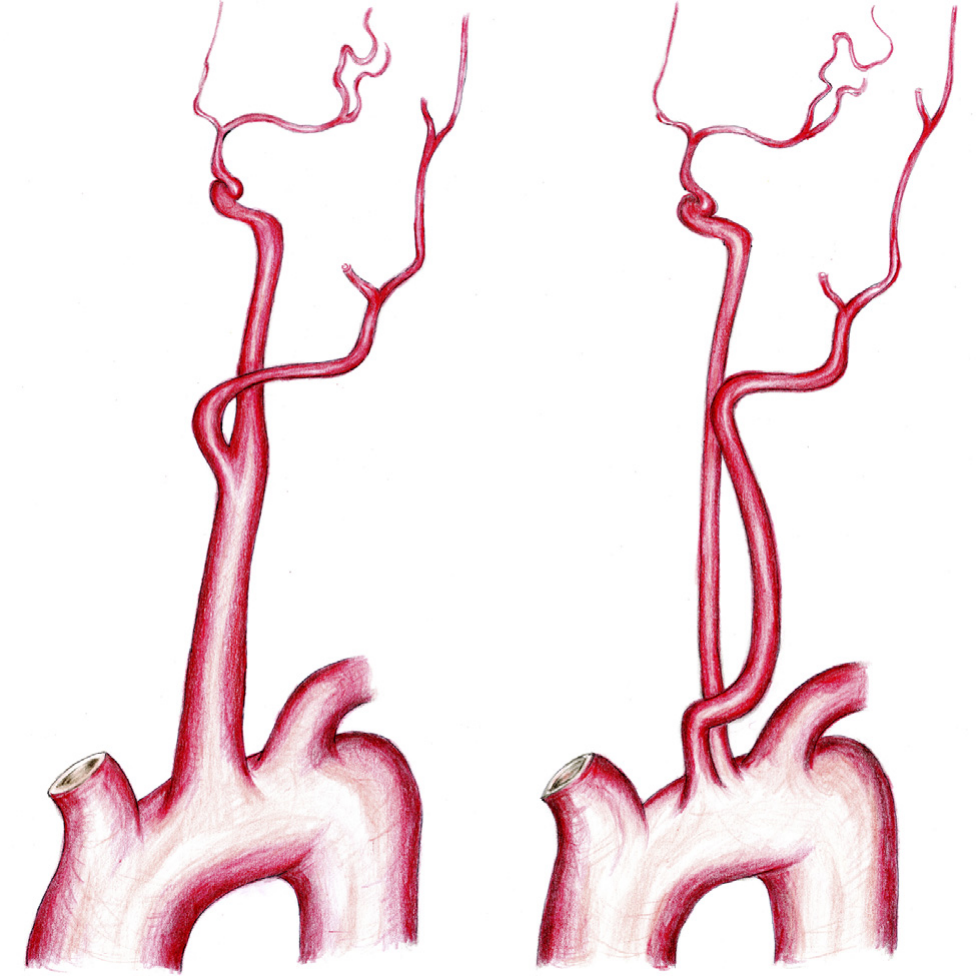
Schematic diagram showing the normal anatomy (left) and the separate origins of the left internal and external carotid arteries from the aorta (right).

We report the case of a patient with separate origins of the left internal and external carotid arteries from the aorta with right intracerebral hemorrhage. Agenesis of the left common carotid artery with separate origins of the left internal and external carotid arteries from the aorta is a very rare anomaly (Fig. 4). This anomaly is usually incidentally found at autopsy or during workup for other clinical problems [1]. There are no sex-related or left–right differences, and bilateral cases have been reported, with approximately 40 cases reported to date [2,3]. Separate origins of the left internal and external carotid arteries from the aorta are considered to be a developmental abnormality. The absence of a common carotid artery may occur if the ductus caroticus persists and the third aortic arch regresses, or if the third aortic arch persists and the fourth aortic arch regresses [4].

Although most cases of this anomaly are asymptomatic, there have been reports of symptomatic lesions; Bryan *et al.* reported a patient who presented with transient ischemic attack [5]. In that case, the left ICA was hypoplastic, tortuous, and kinked near its origin. It is thought that the low perfusion of the left carotid circulation caused the left hemisphere ischemia. In our case, the left ICA was also tortuous and smaller than the left ECA and the right ICA (Fig. 2). Angiography also revealed crossflow via the anterior communicating artery (Fig. 3). Considering the hypoperfusion that was present in the previously reported case, there may also have been hemodynamic stress on the contralateral (right) side in our case. Warschewske *et al.* reported a case of contralateral giant ICA aneurysm associated with separate origins of the left internal and external carotid arteries from the aortic arch [6]. This case also suggested that hemodynamic stress to the contralateral (right) side was involved. In the present case, we believe that the separate origins of the left internal and external carotid arteries could have caused hemodynamic stress on the contralateral side, leading to right intracerebral hemorrhage.

## Conclusion

Here, we report a case of a patient with separate origins of the left internal and external carotid arteries from the aorta and intracerebral hemorrhage. Although this anomaly is typically asymptomatic, it may cause hemodynamic stress on the contralateral side, leading to intracerebral hemorrhage.

## Patient Consent Statement

This study was approved by the institutional review board, and informed consent was obtained from the patient.
